# Microstructure and Corrosion Behavior of (CoCrFeNi)_95_Nb_5_ High-Entropy Alloy Coating Fabricated by Plasma Spraying

**DOI:** 10.3390/ma12050694

**Published:** 2019-02-27

**Authors:** Wenrui Wang, Wu Qi, Lu Xie, Xiao Yang, Jiangtao Li, Yong Zhang

**Affiliations:** 1 School of Mechanical Engineering, University of Science and Technology Beijing, Beijing 100083, China; gmbitwrw@ustb.edu.cn (W.W.); wu.qi@xs.ustb.edu.cn (W.Q.); 2 Key Laboratory of Fluid Interaction with Material, Ministry of Education, Beijing 100083, China; 3 Key Laboratory of Cryogenics, Technical Institute of Physics and Chemistry, Chinese Academy of Sciences, Beijing 100190, China; lijiangtao@mail.ipc.ac.cn; 4 Center of Materials Science and Optoelectronics Engineering, University of Chinese Academy of Sciences, Beijing 100049, China; 5 State Key Laboratory for Advanced Metals and Materials, University of Science and Technology Beijing, Beijing 100083, China; drzhangy@ustb.edu.cn

**Keywords:** high-entropy alloy coating, plasma spray, microstructure, corrosion resistance

## Abstract

In this paper, the (CoCrFeNi)_95_Nb_5_ high-entropy alloy (HEA) coating with a thickness of 500 μm on Q235 steel substrate was fabricated by plasma spraying. The microscopic results showed that a new Laves phase is formed in the (CoCrFeNi)_95_Nb_5_ HEA coating compared to the HEA powder, and elemental segregation occurs between the dendrites and the interdendrites of the coating, while the interdendritic phase enriches with the Cr and Nb. The phase composition change and elemental segregation behavior were mainly due to the faster cooling rate of the plasma spraying technique. At the junction of the coating and the substrate, the HEA coating bonded well to the substrate; in addition, the width of transition zone was merely 2 μm. The microhardness of the (CoCrFeNi)_95_Nb_5_ HEA coating was 321 HV0.5, which is significantly higher than that of the substrate. In terms of corrosion resistance, the (CoCrFeNi)_95_Nb_5_ HEA coating has good corrosion resistance in NaCl solution. Although the corrosion form was pitting corrosion, the pitting potential of the (CoCrFeNi)_95_Nb_5_ HEA coating was significantly higher than that of other coatings, which was mainly because of the dense passivation film formed by Cr and Nb on the surface of the coating. Once the passivation film was destroyed by Cl^−^, the selective corrosion occurred on the surface of the (CoCrFeNi)_95_Nb_5_ HEA coating. In summary, the (CoCrFeNi)_95_Nb_5_ HEA coating prepared by plasma spraying technology can significantly improve the corrosion resistance and mechanical properties of the Q235 steel substrate.

## 1. Introduction

In the past decade, high-entropy alloys (HEAs), containing at least four principle elements in equimolar or near-equimolar ratios, have received more and more attention due to the remarkable comprehensive performance. Unlike conventional alloys materials based on one or two principal constituent elements, HEAs tend to form a simple multi-component solid solution structure, such as face-centered cubic (FCC) structure, body-centered cubic (BCC) structure, and hexagonal close-packed (HCP) structure [1,2,3,4,5].

Among these, CoCrFeNi system HEAs, which possess the typical FCC solid solution structure, exhibit a remarkable combination of high ductility, cryogenic fracture toughness, corrosion resistance, and thermal stability. Moreover, such FCC-typed HEAs have been proven to achieve high strength while maintaining sufficient ductility by means of precipitation strengthening mechanisms. For instance, He et al. developed high-performance HEAs with nano-sized coherent precipitates, L1_2_-Ni_3_(Ti, Al), in CoCrFeNi matrix by adding a small amount of Ti and Al elements [6]. Liu et al. confirmed that the precipitation of ordered Nb-enriched Laves phase can effectively enhance the strength of CoCrFeNiNb_x_ HEAs [7]. Thus, CoCrFeNi system HEAs are considered a potential high-performance structural material.

However, compared to conventional Ni-based superalloys and steel, high density (~8 g/cm^3^) and cost severely limit its engineer application. This is caused by the expensive metal elements used, such as Co and Ni, for bulk CoCrFeNi system HEAs. At present, a recognized and effective solution is to develop high performance HEAs coatings on substrates of traditional materials, which could give full play to its performance advantages, and an increasing number of work on HEAs coating have been reported [8,9,10,11,12].

Traditional HEAs coating preparation methods are mainly focused on laser cladding [8,9], laser metal deposition [10], mechanical alloying, and hot pressing sintering [11,12]. Different from these technologies, the plasma spray process has more advantages: (1) the ingredient of coating is easy to control precisely; (2) the temperature rise of the substrate is small and does not affect the shape and performance of the substrate. Therefore, plasma spraying technology has great application potential for preparing HEA coatings with excellent performance.

In this work, (CoCrFeNi)_95_Nb_5_ HEA were selected as the composition of coating because of its high strength and corrosion resistance in bulk state, while the corresponding properties of coating remain unknown. Therefore, (CoCrFeNi)_95_Nb_5_ HEA coating on the Q235 steel substrate was fabricated by using the plasma spray technique, and the microstructure evolution during preparation process, microhardness, and corrosion behavior in 3.5 wt.% NaCl solution were investigated. Furthermore, the phase formation and the corrosion behavior of the coatings were specifically analyzed.

## 2. Experimental Procedures

The Q235 steel was used as the substrate, and its chemical composition was Fe (base), C (≤0.22%), Si (≤0.35%), Mn (≤1.4%), P (≤0.045%), and S (≤0.05%). First, surface impurities were removed by acetone in an ultrasonic cleaner. Metal powder of Co, Cr, Fe, Ni, and Nb with purity more than 99.9% was used to prepare (CoCrFeNi)_95_Nb_5_ HEA powder by atomization method. Then, the (CoCrFeNi)_95_Nb_5_ HEA powder was used as raw material to prepare HEA coating by vacuum plasma spraying system (ZP-2000, DWAUTO, Xi’an, China). Plasma spraying process parameters: arc power was 30 kW, current was 500 A, main gas Ar flow was 45 L/min, and powder delivery gas flow was 4 L/min; the spraying distance was 100 mm.

The crystal structures of the HEA powder and coating were identified by X-ray diffractometer (XRD, Rigaku D/MAX-RB, Rigaku Corporation, Tokyo, Japan) using Cu-Kα radiation scanning from 20° to 100° at a scanning rate of 10°/min. The microstructure and chemical composition of the HEA powder and coating were observed by scanning electron microscope (SEM, ZEISS SUPRA 55, ZEISS, Oberkochen, Germany) coupled with energy dispersive spectrometry (EDS). The microhardness measurements of HEA coatings at different depths were tested by digital micro Vickers (401MVD, WOLPERT Co., Norwood, MA, USA) under a load of 500 g for 15 s. For each depth, 10 repetitions were performed. After removing the maximum and minimum values, the average value was calculated.

The electrochemical polarization curve of the (CoCrFeNi)_95_Nb_5_ HEA coating was investigated by the electrochemical workstation (Versa STAT MC, PARSTAT 4000, AMETEK Co., Princeton, NJ, USA) in the 3.5 wt.% NaCl solution open to air at room temperature of 298 K. A three-electrode system was used for the electrochemical test, in which the working electrode was the HEA coating sample. The platinum plate was used as an auxiliary electrode, and the reference electrode was a saturated calomel electrode (SCE) with E = 0.244 V_SHE_. The potential scanning range was −0.5 to 0.5 V, and the scan rate was 1 mV/s. After the electrochemical test, the sample was washed with deionized water in an ultrasonic cleaner and dried. Subsequently, the morphology and chemical composition of the sample was observed by SEM coupled with EDS.

An X-ray photoelectron spectrometer (XPS, ESCALAB 250Xi, Thermo Fisher Scientific, Waltham, MA, USA) was employed to study the oxide phases formed on the HEA coating surface, and the XPS spectra were analyzed by Thermo Avantage 5.9 software.

## 3. Results

### 3.1. Microstructure of (CoCrFeNi)_95_Nb_5_ HEA Powder

**Figure 1:**
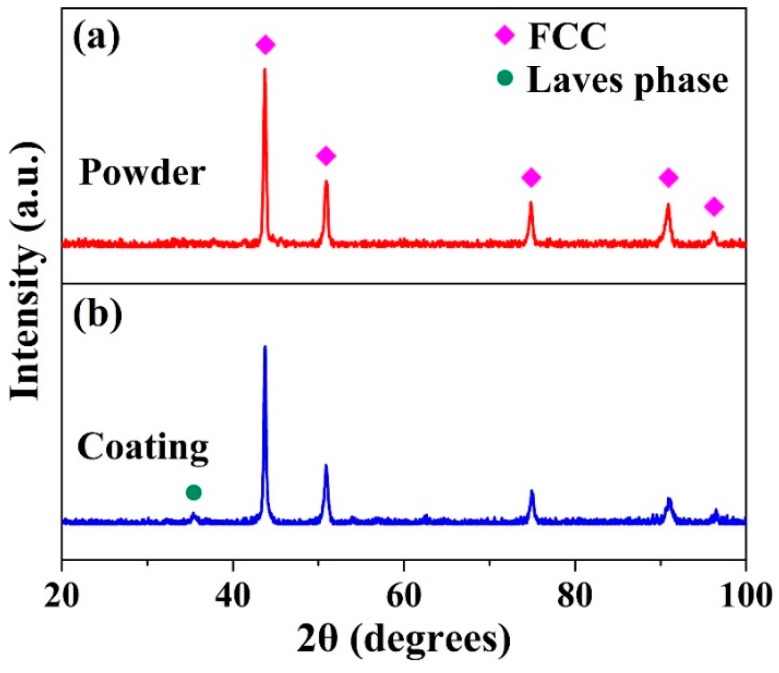
X-ray diffractometer (XRD) patterns of (CoCrFeNi)_95_Nb_5_ high-entropy alloy (HEA) powder (**a**) and coating (**b**).

The XRD pattern of the (CoCrFeNi)_95_Nb_5_ HEA powder is shown in Figure 1a. As can be seen, only diffraction peak of simple FCC phase is found on the pattern, indicating no precipitation of second phase. This result is significantly different from the as-cast CoCrFeNiNb_x_ HEAs with similar composition, where the ordered Laves phase with hexagonal close-packed (HCP) structure could form in FCC matrix with additions of a small amount of Nb. Obviously, during the preparation process, the second phase is inhibited and the supersaturated solid solution structure in high temperature state is retained to room temperature during atomization process.

**Figure 2:**
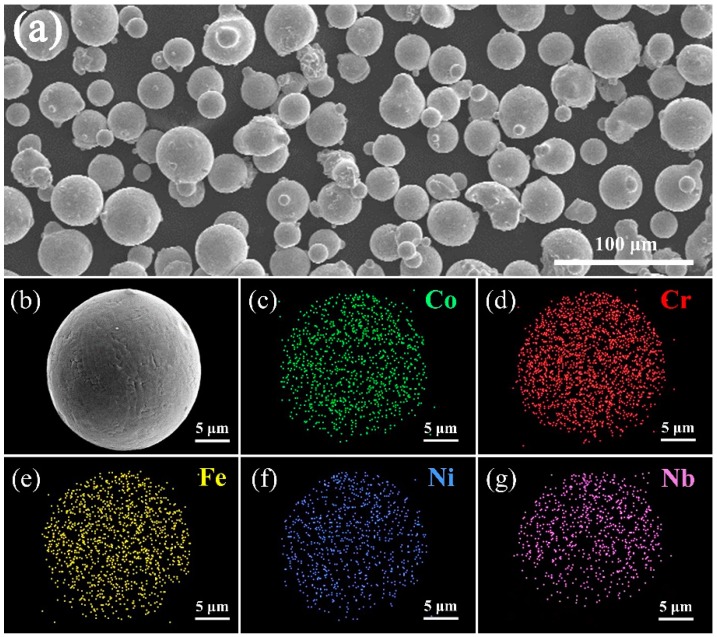
(**a**,**b**) Scanning electron microscope (SEM) images of the (CoCrFeNi)_95_Nb_5_ HEA powder, (**c**–**g**) Chemical analysis of HEA powder by energy dispersive spectrometry (EDS).

The SEM image of (CoCrFeNi)_95_Nb_5_ HEA powder is shown in Figure 2a. It can be seen that the powder is nearly spherical, and the diameter of the particles is from 10 μm to 30 μm. As seen from Figure 2b, the surface of the spherical powder is not smooth, distributing inhomogeneously as a grid. The EDS results (Table 1) and mappings (Figure 2c–g) of HEA powder indicate that composite of powder is very close to the designed nominal composition and the distribution of each element in the single powder is highly uniform.

### 3.2. Microstructure of (CoCrFeNi)_95_Nb_5_ HEA Coating

Using the above HEA powder, the HEA coating was successfully prepared by plasma spraying. Figure 1b shows the XRD pattern of the HEA coating. Compared to the XRD pattern of the HEA powder, there was one minor diffraction peak determined as the Laves phase on the pattern at about 36°, which is consistent with the phase constituent of as-cast CoCrFeNiNb_x_ HEAs reported by Jiang et al. [13], and the peak intensity of the Laves phase was significantly lower than that of the FCC phase, indicating the FCC phase is the main phase of the alloy.

**Figure 3:**
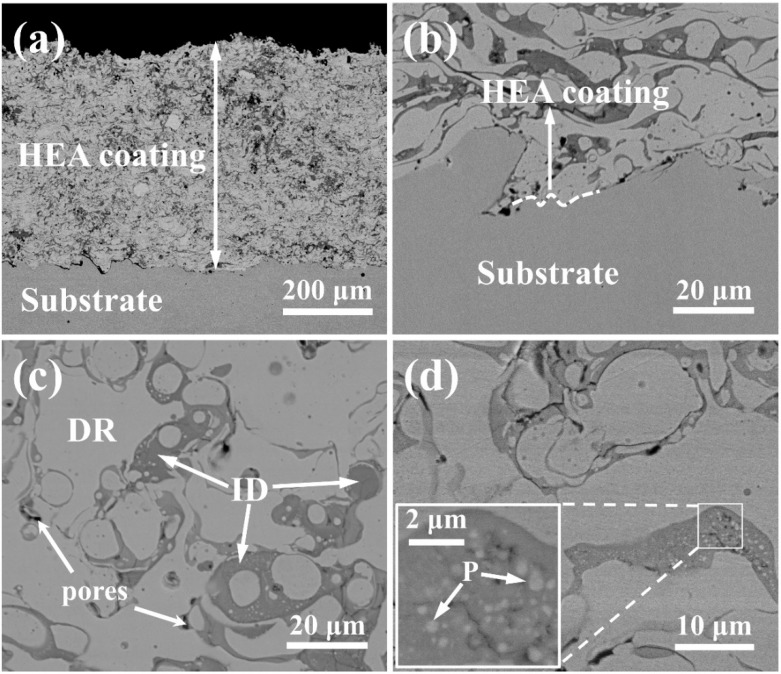
SEM images of (CoCrFeNi)_95_Nb_5_ HEA coating: (**a**) longitudinal section, (**b**) high magnification morphology of longitudinal section, (**c**) coating surface, (**d**) high magnification morphology of the coating surface. DR: dendrites with white contrast; ID: interdendrites with gray contrast.

Figure 3a exhibits the low magnification morphology of longitudinal section, illustrating that the thickness of the coating on the Q235 steel substrate was approximately 500 μm and that the surface of the coating was uneven. Figure 3b is an enlarged view of the longitudinal section of the bonding surface between the HEA coating and the substrate. It can be seen that there is a clear interface between the coating and the substrate; however, the combination between the two parts was tight and only a small amount of pores was observed. The longitudinal section of the coating shows a distinct elongated dendritic and interdendritic structure parallel to the surface of the substrate, which exhibits the traces of expansion and subsequent solidification of the melt HEAs droplets on the substrate during plasma spraying.

Figure 3c presents the microstructure of cross section of the (CoCrFeNi)_95_Nb_5_ HEA coating. It was found that the dendrites with white contrast (marked as DR) and interdendrites with gray contrast (marked as ID) show an approximately equiaxed morphology, and black areas are pores. The chemical composition of the dendritic phase and the interdendritic phase was analyzed by EDS, and the results are shown in Table 1. It has been demonstrated that Cr and Nb elements are enriched in the interdendritic phase, while Co, Fe and Ni are mainly concentrated in the dendritic region. Similar composition segregation phenomena have also appeared in the study of Liu et al. [7]. It is noteworthy that a lot of rounded precipitates (marked as P) are found in the gray phases, as shown in the inset of Figure 3d and the corresponding elemental composition was analyzed by EDS. From Table 1, the precipitated phase is rich in Co, Fe, and Nb.

**Figure 4:**
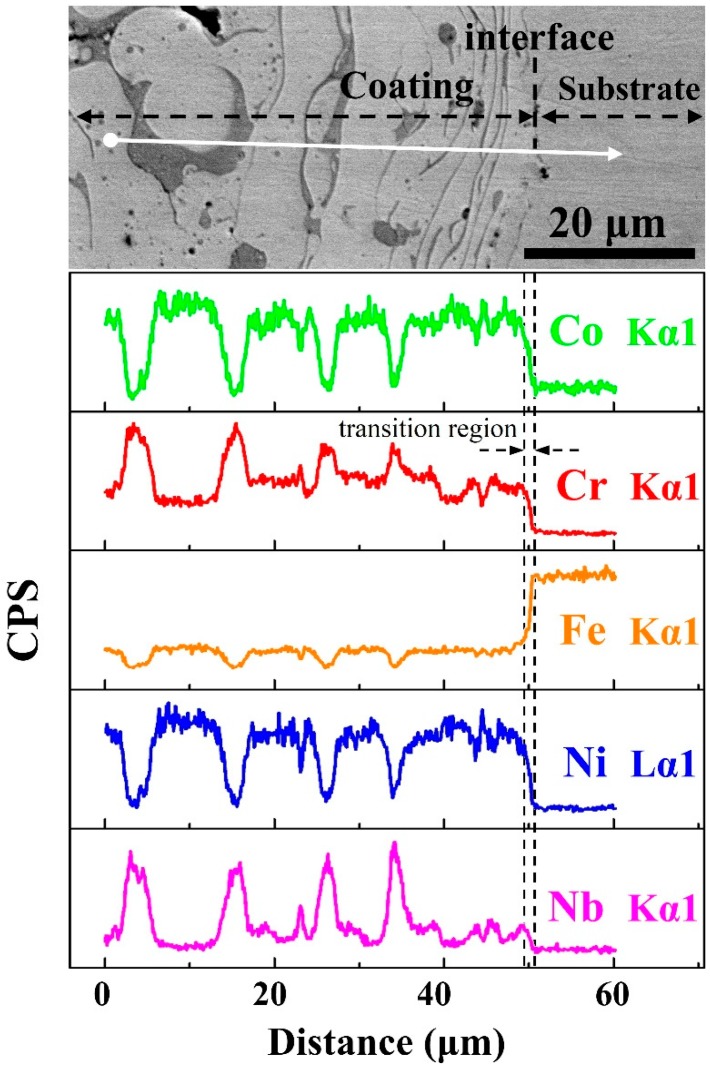
(**a**) SEM image of the HEA coating longitudinal section, (**b**–**f**) distributions of alloying elements by EDS line scanning.

In order to analyze the elemental partitioning behavior across the coating-substrate interface, the EDS line scan performs on the white line on Figure 4a, and the distributions of different alloying elements are shown in Figure 4b–f. Elemental composition does not sharply change at the interface; however, there is a distinct diffusion transition zone with a width of about 2 μm between coating and substrate, indicating that the HEA coating and Q235 steel substrate are combined by metallurgical bonding, and the heat-affected zone is narrow during plasma spraying process.

### 3.3. Microhardness of the (CoCrFeNi)_95_Nb_5_ HEA Coating

**Figure 5:**
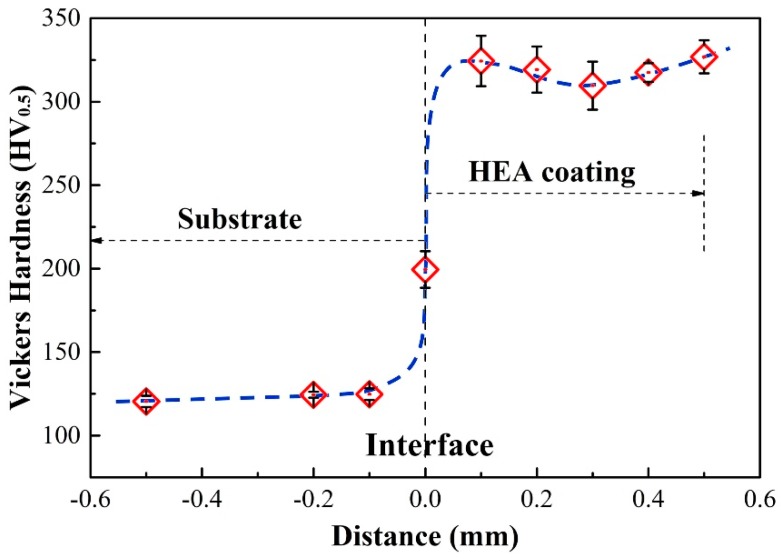
Microhardness distribution of (CoCrFeNi)_95_Nb_5_ HEA coating.

In order to investigate the mechanical behavior of HEAs coating, the microhardness varying from the substrate to the surface of the (CoCrFeNi)_95_Nb_5_ HEA coating is tested as shown in Figure 5. The results show that the microhardness of the HEA coating, 310~325 HV0.5, is significantly higher than that of the Q235 steel substrate, ~122 HV0.5, and the hardness of the interface is between such two parts. The change trend of microhardness corresponds to that of microstructure and composition, as shown in Figure 3 and Figure 4. It means that HEAs coating with high microhardness, which is attributed to the solution strengthening caused by the high size mismatch between Nb with Co, Cr, Fe, and Ni, and the precipitation strengthening caused by the precipitation of the ordered Laves phase, can effectively improve the comprehensive mechanical property of the substrate.

### 3.4. Corrosion Performance of (CoCrFeNi)_95_Nb_5_ HEA Coating

**Figure 6:**
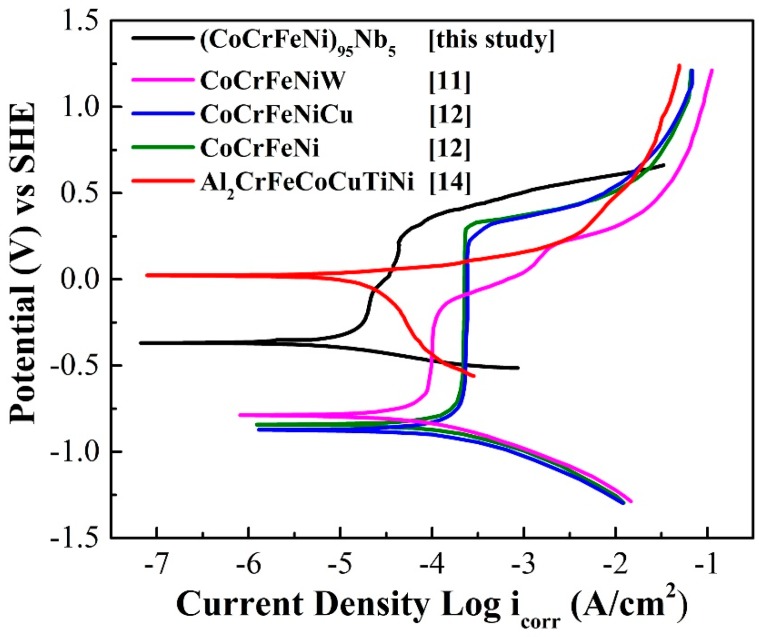
Polarization curves of (CoCrFeNi)_95_Nb_5_ HEA coating and the reported HEA coatings in 3.5 wt.% NaCl solution.

The polarization curve of (CoCrFeNi)_95_Nb_5_ HEA coating in 3.5 wt.% NaCl solution is shown in Figure 6. For comparison, Figure 6 also exhibits the polarization curves of other HEA coatings prepared by other processes in 3.5 wt.% NaCl solution, and the corrosion dynamics parameters of all HEA coatings obtained by linear fit are given in Table 2. As can be seen, the (CoCrFeNi)_95_Nb_5_ HEA and Al_2_CrFeCoCuTiNi HEA coatings have higher corrosion potential than other coatings, and the (CoCrFeNi)_95_Nb_5_ HEA coating also possesses the lowest corrosion current density, indicating that the (CoCrFeNi)_95_Nb_5_ HEA coating has excellent comprehensive corrosion resistance in 3.5 wt.% NaCl solution. Furthermore, although the pitting potential of (CoCrFeNi)_95_Nb_5_ HEA coating is similar to that of other CoCrFeNi-based coating, the lower passivation current density of (CoCrFeNi)_95_Nb_5_ HEA coating means the better pitting resistance.

**Figure 7:**
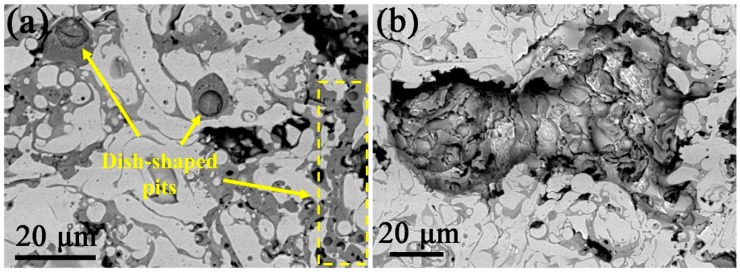
SEM images of corrosion morphology after electrochemical test: (**a**) dish-shaped pitting pits, (**b**) a large corrosion pit.

Figure 7 shows the SEM images of the surface of the (CoCrFeNi)_95_Nb_5_ HEA coating after electrochemical experiment in 3.5 wt.% NaCl solution. Obviously, a large number of tiny dish-shaped pitting pits with diameter of 1~5 μm are observed on the surface of the coating (Figure 7a), and such pitting pits are only distributed in gray phase region. Figure 7b presents a large corrosion pit where many secondary pitting holes are formed on the inner wall of the pit. Furthermore, those pitting holes are mainly displayed in the interdendritic region; however, the dendritic phase is undamaged, indicating that selective corrosion occurs on the surface of the HEA coating. According to the foregoing EDS results (listed in Table 1), it can be inferred that in the 3.5 wt.% NaCl solution, the interdendritic phase rich in Nb and Cr is more easily corroded, while the dendritic phase rich in Co, Ni, and Fe is less susceptible to corrosion.

## 4. Discussion

### 4.1. Phase Formation

According to the phase analysis results obtained above, it was found that, although having the same chemical composition, the phase composition of the sample changed significantly at different preparation stages. That is, the ordered Laves phase is suppressed in (CoCrFeNi)_95_Nb_5_ HEA powder, and only a single FCC solid solution phase is formed. However, in the coating, the thermodynamically stable ordered Laves phase could precipitate out of the FCC solid solution matrix during plasma spraying process. This indicates that in addition to thermodynamic factors, kinetic factors play a very important role in the process of phase formation.

The results of research on as-cast CoCrFeNiNb_x_ alloys have proven that Nb tends to form ordered phases with other elements because of negative mixing enthalpy, and with increment of Nb, the proportion of Laves phase increases in the alloy [17,18]. However, the effect of mixing enthalpy on phase formation during coating preparation is not significant. The mixing enthalpy between the elements of the high-entropy alloy is shown in Table 3. The mixed enthalpy of Nb and Cr is larger than the other three elements, indicating that Nb has a relatively weak binding force with Cr; however, the actual situation is that Nb and Cr are enriched in the interdendritic phase. This phenomenon may be due to the difference in melting point of the element and the characteristics of the plasma spraying process. The melting points of Cr and Nb are 2133 K and 2750 K, respectively, significantly higher than Co (1770 K), Fe (1811 K) and Ni (1728 K). Due to the rapid solidification of plasma spraying, the Cr and Nb-rich phases with high melting point have solidified before they converted into stable phases, which leads to the enrichment of Cr and Nb elements in the interdendritic phase. In addition, a small amount of the Co- and Fe-saturated precipitates formed in the interdendritic region as shown in the new phase in Figure 3d, which are rich in Co, Fe and Nb, and have better thermodynamic stability than interdendritic phase. This indicates that the interdendritic phase tends to shift to a more stable morphology.

### 4.2. Corrosion Resistance

**Figure 8:**
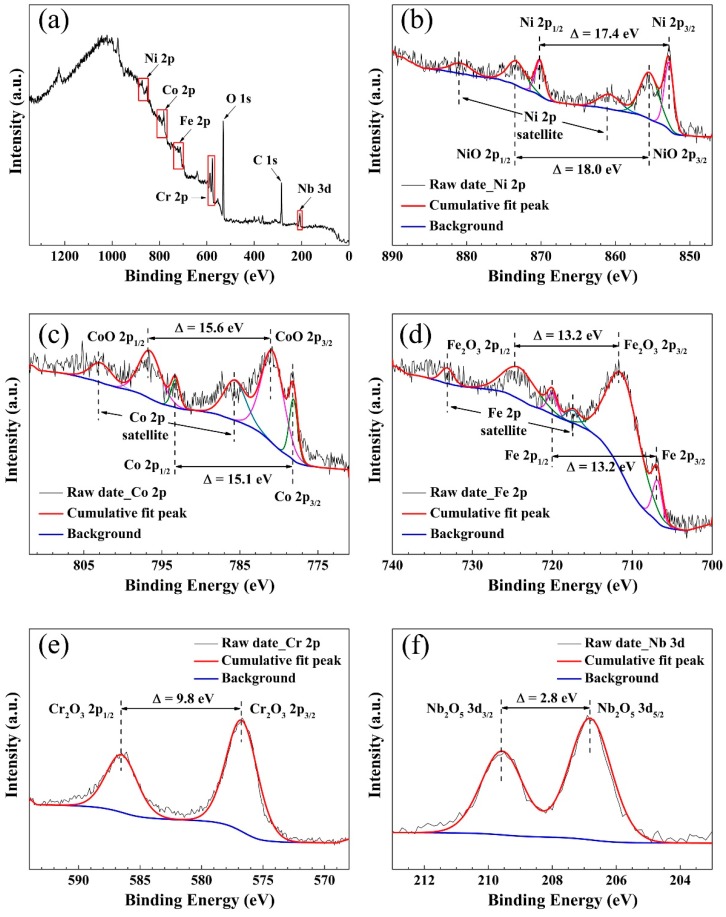
X-ray photoelectron spectrometer (XPS) spectra of the passivation film on (CoCrFeNi)_95_Nb_5_ HEA coating (**a**) full survey, (**b**) Ni 2p, (**c**) Co 2p, (**d**) Fe 2p, (**e**) Cr 2p, (**f**) Nb 3d.

Electrochemical experiments have demonstrated that the (CoCrFeNi)_95_Nb_5_ HEA coating exhibits excellent corrosion resistance, and that the passivation film of the coating may play an important role. To prove this guess, chemical compositions of the passive film formed on the surface of the (CoCrFeNi)_95_Nb_5_ HEA coating after air oxidation were investigated by XPS. Figure 8 shows the XPS spectra of the passivation film on the surface of HEA coating in air. The full survey spectrum (Figure 8a) exhibits that the passivation film contains seven elements of Co, Cr, Fe, Ni, Nb, O, and C [19]. The peaks of Ni 2p correspond to Ni and NiO (Figure 8b). The Co 2p peaks are determined as Co and CoO (Figure 8c). The peaks of Fe 2p are identified as Fe and Fe_2_O_3_ (Figure 7d). The Cr 2p peaks correspond to Cr_2_O_3_ (Figure 8e). Lastly, Nb_2_O_5_ (Figure 8f) is observed in the peaks of Nb 3d.

Early research reported that stainless steel, Fe-Cr alloy, and CrFe_15_Ni_15_ alloy have excellent corrosion resistance due to the dense Cr_2_O_3_ passivation film formed on the surface [20,21,22]. Similarly, it was speculated that the Cr_2_O_3_ passivation film has the same effect in the corrosion resistance of HEAs. AlCoCrFeNi [23], Al_x_CrFe_1.5_MnNi_0.5_ [24], and Al_0.5_CoCrCuFeNiB_x_ [25] verified the foregoing speculation. Therefore, for the (CoCrFeNi)_95_Nb_5_ HEA coating passivation film of this study, it can be obtained from the results of XPS that the Cr_2_O_3_ in the passivation film provides good corrosion resistance for the coating, and the addition of trace amount of Nb results in forming a stable metal oxide of Nb_2_O_5_ in the passivation film of the coating, which also plays a positive role in the corrosion resistance [26,27].

Nevertheless, although the passivation film can improve the corrosion resistance, the (CoCrFeNi)_95_Nb_5_ HEA coating will inevitably be subjected to some local corrosion as with other corrosion-resistant alloys. Owing to the more negative the potential of the metal standard electrode, the easier it is to lose electrons and dissolve. Moreover, for the alloy of this paper, the standard potential of Nb (−1.1 V) and Cr (−0.74 V) is significantly lower than that of Fe (−0.44 V), Ni (−0.25 V) and Co (−0.28 V) [28,29]. Therefore, during the electrochemical experiment, once the local passivation film is destroyed, the interdendritic phase rich in Nb and Cr is preferentially corroded as an anode, and the dendritic phase rich in Co, Ni, and Fe is protected as a cathode, which results in selective corrosion on the surface of the (CoCrFeNi)_95_Nb_5_ HEA coating. Furthermore, as the pitting behavior continues, the adjacent interdendritic regions are completely corroded, causing catastrophic corrosion (Figure 7b). Similarly, this phenomenon of selective corrosion was observed in CrMnFeCoNi HEA [30] and AlCoCrFeNi [31].

## 5. Conclusions

(1) The (CoCrFeNi)_95_Nb_5_ HEA coating was prepared by plasma spraying on substrate of the Q235 steel, consisting of a simple FCC solid solution with Laves phase, and the coating bonded well to the substrate.

(2) Elemental segregation occurred between the dendrites and the interdendrites of the coating, and the elements Cr and Nb with high melting point were enriched between the interdendrites. This phenomenon may be due to the difference in melting point of the element and the characteristics of the plasma spraying process.

(3) The coating has a microhardness of 321 HV0.5, which can effectively improve the surface hardness of the Q235 steel part.

(4) The (CoCrFeNi)_95_Nb_5_ HEA coating exhibited excellent corrosion resistance in the electrochemical corrosion test of a 3.5 wt.% NaCl solution comparing to previously studied coatings prepared by other processes.

(5) In the 3.5 wt.% NaCl solution, the selective corrosion occurred on the surface of (CoCrFeNi)_95_Nb_5_ HEA coating, while the interdendritic phase rich in Nb and Cr was preferentially corroded as an anode, and the dendritic phase rich in Co, Ni, and Fe was protected as a cathode.

## Figures and Tables

**Table 1:** Nominal compositions and chemical compositions of (CoCrFeNi)_95_Nb_5_ HEA powder and coating (at.%).

Sample		Co	Cr	Fe	Ni	Nb
Nominal Composition		23.75	23.75	23.75	23.75	5.00
Powder		23.74	24.16	23.91	23.23	4.95
Coating	Dendrite	27.28	19.03	25.95	25.71	2.03
	Interdendrite	5.05	59.91	12.48	3.80	18.76
	Precipitate	34.62	16.78	26.42	11.29	10.89

**Table 2:** Corrosion dynamics parameters of different HEA coatings in 3.5 wt.% NaCl solution.

Coating	Method	*i_corr_*^a^ (A·cm^−2^)	*E_corr_*^b^ (V)	*E_pit_*^c^ (V)	*i_pass_*^d^ (A·cm^−2^)	*ΔE*^e^ (V)	References
(CoCrFeNi)_95_Nb_5_	Plasma spraying	7.23 × 10^−6^	−0.37	0.26	4.13 × 10^−5^	0.63	[this study]
CoCrFeNiW	MA+HPS ^f^	1.42 × 10^−5^	−0.78	−0.16	1.01 × 10^−4^	0.62	[11]
CoCrFeNiCu		1.77 × 10^−5^	−0.84	0.30	2.43 × 10^−4^	1.14	[12]
CoCrFeNi		9.44 × 10^−6^	−0.87	0.33	2.25 × 10^−4^	1.20	[12]
Al_2_CrFeCoCuTiNi	Laser cladding	1.30 × 10^−5^	0.02	- ^g^	- ^g^	- ^g^	[14]

^a^*i_corr_*: corrosion current density; ^b^*E_corr_*: corrosion potential; ^c^*E_pit_*: pitting potential; ^d^*i_pass_*: passive current density; ^e^*ΔE*: E_pit_ − E_corr_; ^f^ MA + HPS: mechanical alloying and hot pressing sintering; ^g^ the passivation region is nearly non-existent, is not obvious, or can be ignored.

**Table 3:** Mixing enthalpy (*ΔH_mix_*, kJ/mol) values of atom pairs and melting temperature of component element (*T_m_*, K) in (CoCrFeNi)_95_Nb_5_ HEA coating [15,16].

	Co	Cr	Fe	Ni	Nb
Cr	−4	*	*	*	*
Fe	−1	−1	*	*	*
Ni	0	−7	−2	*	*
Nb	−25	−7	−16	−30	*
*T_m_*	1770	2133	1811	1728	2750
